# Supplementary material for: Target guided synthesis using DNA nano-templates for selectively assembling a G-quadruplex binding c-MYC inhibitor
Source: Nat Commun. 2017 Jul 14;8:16103. doi: 10.1038/ncomms16103 (PMC5519986; doi:10.1038/ncomms16103)
Supplement: Supplementary Information [file ncomms16103-s1.pdf]

SI GUIDE

Title of file for HTML: Supplementary Information
Description: Supplementary Figures, Supplementary Methods and Supplementary References.

Title of file for HTML: Peer Review File
Description:

Supplementary Methods

General information: All solvents and reagents were purified by standard techniques reported in Armarego, W. L. F., Chai, C. L. L., Purification of Laboratory Chemicals, 5th edition, Elsevier, 2003; or used as supplied from commercial sources (Sigma-Aldrich Corporation® unless stated otherwise). All reactions were generally carried out under inert atmosphere unless otherwise noted. TLC was performed on Merck Kieselgel 60 F254 plates, and spots were visualized under UV light. Products were purified by flash chromatography on silica gel (100-200 mesh, Merck). ^{1}H and ^{13}C NMR spectra were recorded on either Brüker ADVANCE 500 (500 MHz and 125 MHz), or JEOL 400 (400 MHz and 100 MHz) instruments using deuterated solvents as detailed and at ambient probe temperature (300 K). Chemical shifts are reported in parts per million (ppm) and are referred to the residual solvent peak. The following notations are used: singlet (s); doublet (d); triplet (t); quartet (q); multiplet (m); broad (br). Coupling constants are quoted in Hertz and are denoted as *J*. Mass spectra were recorded on a Micromass® Q-Tof (ESI) spectrometer.

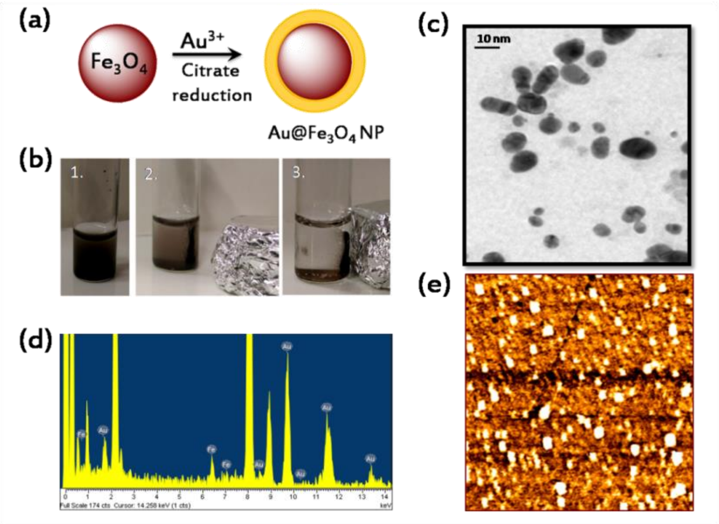

Supplementary Figure 1. Characterization of Au@Fe_3O_4 nanoparticles. (**a**) Preparation of Au@Fe_3O_4 nanoparticle by citrate reduction. (**b**) DNA linked Au@Fe_3O_4 nanoparticle solutions showing good para-magnetic property; 1. The nanoparticles are well dispersed in Tris.KCl buffer (pH 7.4) with no magnet in the vicinity, 2. Nanoparticles started to separate under the effect of a magnet 3. nanoparticles are completely separated at the right hand side of the bottle. (**c**) TEM imaging (software Gatan Digital Micrograph) and (**d**) EDX spectrum of Au@Fe_3O_4 nanoparticles. (**e**) AFM image of Au@Fe_3O_4 nanoparticles (software Image Processing and Analysis 3.5.0.2060 program).

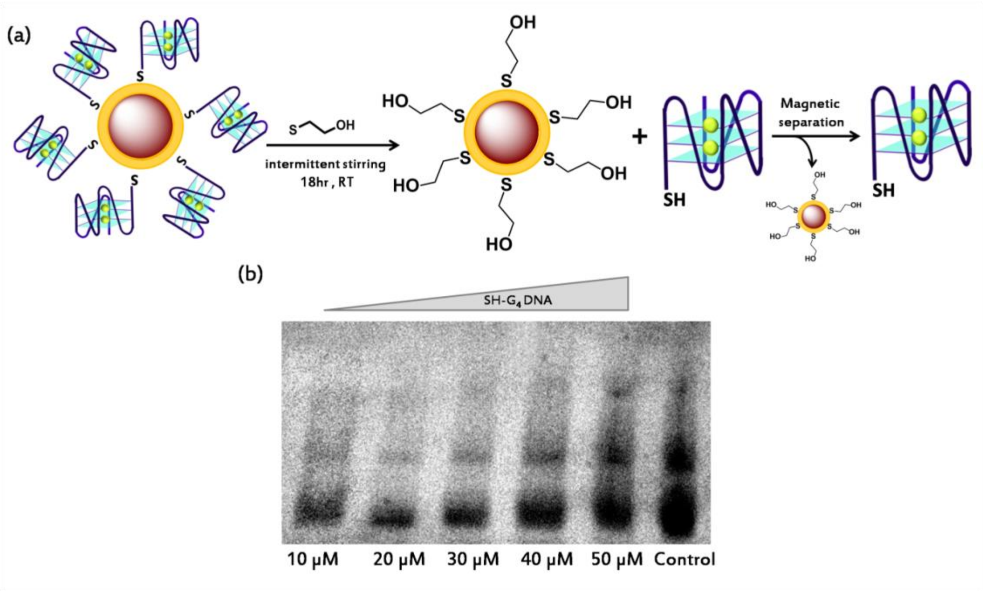

Supplementary Figure 2. Evidence for surface modification of Au@Fe_3O_4-NPs with DNA. (**a**) Schematic representation of the method for the displacement of oligonucleotides from the Au@Fe_3O_4 nanoparticles via β-mercaptoethanol exchange reaction. (**b**) Native PAGE analysis of the displaced oligonucleotides to determine the extent of surface coverage of the nanoparticles by oligonucleotides.

Synthesis of alkyne bulding blocks:

(i) Synthesis of carbazole alkyne 1a: The carbazole alkyne **1a** was prepared from commercially available carbazole **S1** as shown in **Supplementary Figure 3**. The synthesis started with monoiodination of carbazole **S1**, which was carried out using KI and KIO_3 to afford **S2** in 40% yield. The *N*-arylation[1] of **S2** with 4-fluorobenzonitrile **S3** afforded the nitrile derivative **S4** in 92% yield. The basic hydrolysis of nitrile group of **S4** afforded the corresponding acid **S5** in 76% yield. The amide coupling of acid **S5** with 3-(dimethylamino)-propylamine **S6** gave the amide **S7** in 75% yield. Palladium catalyzed sonogashira coupling of **S7** with TMS-acetylene followed by deprotection of sillyl group provided **1a** in 90% yield.

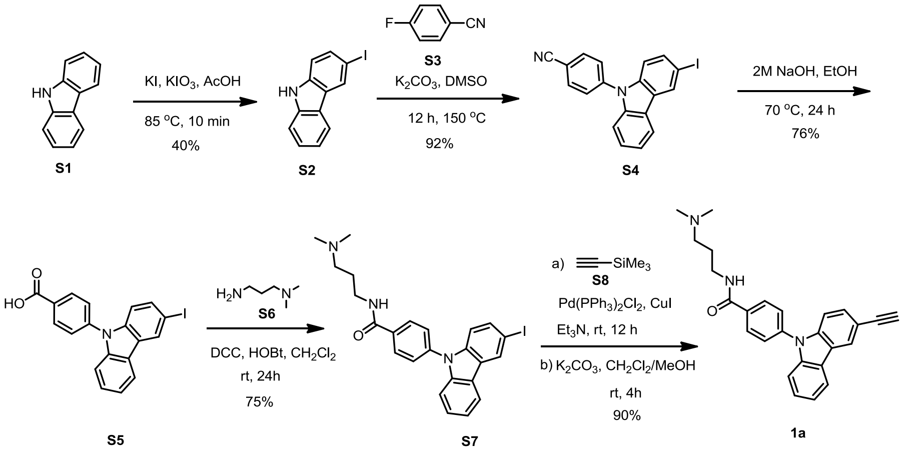

Supplementary Figure 3. Synthesis of alkyne **1a** from commercially available carbazole **S1**.

Preparation of 3-iodocarbazole S2: The iodination of carbazole **S1** was carried out in a 500 mL round-bottom flask equipped with a magnetic stirrer. A mixture of carbazole **S1** (16.7 g, 0.1 mol) and potassium iodide (11.0 g, 0.066 mol) in glacial acetic acid (260 mL) was boiled together at 85 °C. The flask was then cooled and finely powdered potassium iodate (16.0 g, 0.075 mol) was added. The resulting mixture was then stirred at 85 °C for 10 minutes. Then the hot solution was decanted from the undissolved potassium iodate and allowed to cool slowly. The mixture was extracted with ethyl acetate (3 x). The combined organic layers were washed with brine, dried over anhydrous Na_2SO_4 and evaporated under reduced pressure. The residue was purified by column chromatography (hexane/ethyl acetate (50:1) to afford the desired product **S2** (11.7 g, 40%) as a white solid[2].

Preparation of carbazole nitrile S4: In an oven dried round bottom flask fitted with a magnetic stir-bar, a mixture of 3-iodocarbazole **S2** (3.0 g, 10.25 mmol) and K_2CO_3 (2.12 g, 15.38 mmol) in DMSO (20 mL) was stirred at room temperature for 1 h. Then 4-fluorobenzonitrile **S3** (1.49 g, 12.3 mmol) was added portion-wise and the resulting reaction mixture was heated at 150 °C and stirred for 12 h. The mixture was poured into a large amount of ice water and stirred for 1 h and it was extracted with ethyl acetate (3 x). The combined organic layers were washed with brine and then dried over anhydrous $MgSO_4$, filtered and concentrated in vacuo. The residue was purified by column chromatography on silica gel (*n*-hexane/EtOAc, 2:1) to afford the carbazole derivative **S4** (3.72 g, 92%) as a brown solid. R_f = 0.45 (*n*-hexane/EtOAc, 1:1); ^{1}H NMR (500 MHz, DMSO-d^6): δ 8.31 (d, *J* = 7.6, 1H), 8.26 (d, *J* = 7.6, 2H), 8.18-8.16 (m, 3H), 7.73-7.71 (m, 2H), 7.48-7.43 (m, 2H), 7.31-7.28 (m, 1H); ^{13}C NMR (100 MHz, DMSO-d^6): δ 139.8, 138.9, 134.3, 133.3, 129.5, 129.0, 127.1, 126.3, 126.2, 122.9, 120.7, 120.6, 120.3, 112.7, 109.8, 109.7, 83.4; HRMS (ESI) Calcd for $C_{19}H_{11}IN_2$ $[M]^+$: 393.9967, Found 393.9970.

Preparation of carbazole acid S5: The nitrile derivative **S4** (2.9 g, 4.28 mmol) was treated with sodium hydroxide (0.51 g, 12.85 mmol) in ethanol/H_2O (2:1) (20 mL) at 70 °C for 24 h and then acidified with 2 M HCl solution to afford the desired compound **S5** (1.34 g, 76%) as a white solid. ^{1}H NMR (500 MHz, DMSO-d^6): δ 13.21 (s_{br}, 1H), 8.31 (d, *J* = 7.6, 1H), 8.26 (d, *J* = 7.6, 2H), 8.24-8.21 (m, 3H), 7.80-7.75 (m, 2H), 7.50-7.45 (m, 2H), 7.34-7.31 (m, 1H); ^{13}C NMR (100 MHz, DMSO-d^6): δ 166.7, 140.8, 138.8, 134.3, 131.3, 129.0, 127.2, 126.4, 126.3, 123.1, 121.1, 120.9, 120.5, 112.3, 110.0, 109.8, 83.7; HRMS (ESI) Calcd for $C_{19}H_{12}INO_2$ $[M]^+$: 412.9913, Found 412.9908.

Preparation of carbazole amide S7: A mixture of carbazole acid **S5** (1.28 g, 3.1 mmol), DCC (825.3 mg, 4.0 mmol), HOBt (612.5 mg, 4.0 mmol) in CH_2Cl_2 (10 mL) was cooled at 0 °C and then amine **S6** (0.513mL, 2.8 mmol) was added and the mixture was stirred for 24 h at room temperature. The reaction mixture was then quenched by addition of $NaHCO_3$ solution and then it was extracted with CH_2Cl_2 (3 x). The combined organic layers were washed with water and brine, dried over $MgSO_4$ and the solvent was evaporated under reduced pressure. The crude product was purified by column chromatography on silica gel (CH_2Cl_2/MeOH, 20:1) to give

the compound **S7** (1.04 g, 75%) as a yellow liquid. R_f = 0.35 (CH_2Cl_2/MeOH, 15:1); ^{1}H NMR (500 MHz, DMSO-d^6): δ 8.72 (s, 1H), 8.19 (d, *J* = 8.2, 1H), 8.20-8.15 (m, 5H), 7.72 (d, *J* = 8.2, 2H), 7.55-7.53 (m, 2H), 7.44-7.41 (m, 1H), 3.76-3.73 (m, 2H), 2.72 (t, *J* = 6.3, 2H), 2.54 (6H, merged with DMSO peak), 1.98 (t, *J* = 6.3, 2H); ^{13}C NMR (100 MHz, DMSO-d^6): δ 165.4, 139.9, 138.7, 134.2, 129.2, 129.1, 127.0, 126.3, 126.1, 123.0, 120.7, 120.5, 120.2, 112.1, 109.7, 109.6, 83.4, 54.8, 44.8, 37.8, 26.8; HRMS (ESI) Calcd for $C_{24}H_{25}IN_3O$ $[M+H]^+$: 498.1042, Found 498.1039.

Preparation of alkyne 1a: A mixture of **S7** (248.7 mg, 0.5 mmol), $PdCl_2(PPh_3)_2$ (35.1 mg, 0.05 mmol), and CuI (19.0 mg, 0.1 mmol) in Et_3N (5 mL) was stirred at room temperature for 30 min and then trimethylsilylacetylene **S8** (0.142.4 mL, 1.0 mmol) was added drop-wise. The resulting mixture was stirred under an argon atmosphere for 12 h. The mixture was evaporated to dryness and the resulting crude product was purified by column chromatography to give the corresponding trimethylsilyl alkyne, which was further stirred with 5 equiv. K_2CO_3 in methanol-CH_2Cl_2 solution under an argon atmosphere for 4 h. The mixture was concentrated under vacuum and the resulting crude product was purified by column chromatography on silica gel (CH_2Cl_2/MeOH, 20:1) to give alkyne **1a** as a yellow liquid (178.0 mg, 90%). R_f = 0.36 (CH_2Cl_2/MeOH, 15:1); ^{1}H NMR (500 MHz, $CDCl_3$): δ 8.32 (s, 1H), 8.18-8.16 (m, 3H), 8.14-8.09 (m, 3H), 7.67-7.62 (m, 2H), 7.45-7.39 (m, 2H), 7.31-7.28 (m, 1H), 3.70-3.66 (m, 2H), 3.09 (s, 1H), 2.96 (t, *J* = 6.7, 2H), 2.65 (s, 6H), 2.12 (t, *J* = 6.7, 2H); ^{13}C NMR (75 MHz, $CDCl_3$): δ 166.0, 140.2, 139.4, 133.8,133.2, 130.2, 128.8, 128.6, 126.7, 126.4, 126.0, 123.4, 120.9, 120.2, 113.1, 109.9, 109.6, 82.2, 72.9, 58.4, 40.8, 39.7, 25.3; HRMS (ESI) Calcd for $C_{26}H_{26}N_3O$ $[M+H]^+$: 396.2076, Found 396.2071.

(ii) Preparation of alkyne 1b (3-ethynyl-9H-carbazole): The alkyne building block **1b** was prepared from **S2** by Sonogashira coupling with trimethylsilylacetylene **S8** followed by deprotection of the silyl group (**Supplementary Figure 4**).

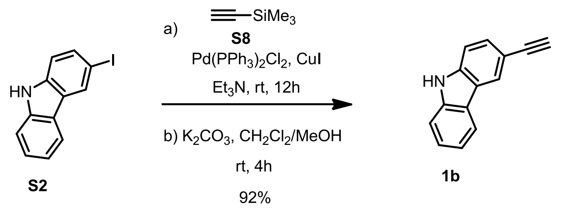

Supplementary Figure 4. Synthesis of alkyne **1b**.

A mixture of iodo carbazole **S2** (146.5 mg, 0.5 mmol), $PdCl_2(PPh_3)_2$ (35.1 mg, 0.05 mmol) and CuI (19.0 mg, 0.1 mmol) in Et_3N (5 mL) was stirred for 30 min at room temperature and then trimethylsilylacetylene **S8** (0.142.4 mL, 1 mmol) was added drop-wise. The resulting mixture was stirred under an argon atmosphere for 12 h at room temperature. The mixture was evaporated to dryness and the resulting crude product was purified by column chromatography to give the corresponding trimethylsilyl alkyne, which was further stirred with 5 equiv. K_2CO_3 in methanol-CH_2Cl_2 solution under an argon atmosphere for 4 h. The mixture was concentrated under

vacuum and the resulting crude product was purified by column chromatography on silica gel (*n*-hexane/EtOAc, 10:1) to give alkyne **1b** as a yellow solid (87.9 mg, 92%). R_f = 0.40 (*n*-hexane/EtOAc, 8:1); ^{1}H NMR (500 MHz, $CDCl_3$): δ 8.15 (s, 1H), 8.07 (d, J = 8.4, 1H), 7.56 (d, J = 8.4, 1H), 7.45-7.44 (m, 3H), 7.37 (d, J = 8.4, 1H), 7.26 (m, 1H), 3.08 (s, 1H); ^{13}C NMR (100 MHz, $CDCl_3$): δ 140.0, 139.5, 130.0, 126.6, 126.0, 124.8, 120.6, 120.5, 120.2, 119.6, 110.9, 110.7, 85.1, 75.3; HRMS (ESI) Calcd for $C_{14}H_9N$ $[M]^+$ 191.0735, Found 191.0731.

(iii) Preparation of alkyne 1c: First, 9-butyl-3-iodocarbazole **S9** was prepared from iodocarbazole **S2** following a literature procedure[2] and then it was used for the preparation of alkyne **1c (Supplementary Figure 5)**. A mixture of iodocarbazole **S9** (174.6 mg, 0.5 mmol), $PdCl_2(PPh_3)_2$ (35.1 mg, 0.05 mmol), and CuI (19.1 mg, 0.1 mmol) in Et_3N (5 mL) was stirred for 30 min at room temperature then trimethylsilylacetylene **S8** (0.142.4 mL, 1 mmol) was added dropwise. The mixture was stirred under an argon atmosphere for 12 h and then evaporated to dryness and the resulting crude product was purified by column chromatography to give the corresponding trimethylsilyl alkyne, which was further stirred with 5 equiv. K_2CO_3 in methanol-CH_2Cl_2 solution under an argon atmosphere for 4 h. The mixture was concentrated under vacuum and the resulting crude product was purified by column chromatography on silica gel (*n*-hexane/EtOAc, 10:1) to give alkyne **1c** as a yellow solid (106 mg, 86%). R_f = 0.42 (*n*-hexane/EtOAc, 9:1); ^{1}H NMR (400 MHz, $CDCl_3$): δ 8.25 (s, 1H), 8.06 (d, J = 7.8, 1H), 7.58 (d, J = 7.3, 1H), 7.46 (d, J = 7.3, 1H), 7.39 (d, J = 8.3, 1H), 7.32 (d, J = 8.3, 1H), 7.26-7.22 (m, 1H), 4.27 (t, J = 6.8, 2H), 3.07 (s, 1H), 1.83 (t, J = 6.8, 2H), 141-1.35 (m, 2H), 0.94 (t, J = 8.4, 3H). ^{13}C NMR (100 MHz, $CDCl_3$): δ 140.9, 140.5, 129.7, 126.3, 125.7, 124.8, 120.6, 120.5, 119.5, 118.8, 109.1, 108.8, 85.3, 75.1, 43.1, 31.2, 20.7, 13.9. HRMS (ESI) Calcd for $C_{18}H_{17}N$ $[M^+]$ 247.1361, Found 247.1355.

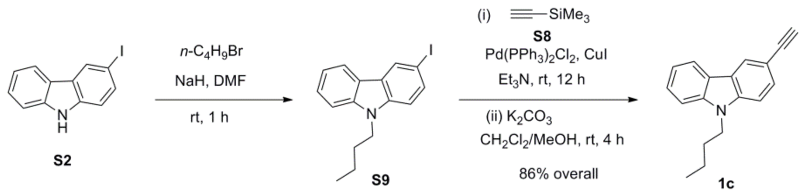

Supplementary Figure 5. Synthesis of alkyne **1c**.

General procedure for G_4•Au@Fe_3O_4 templated azide alkyne cycloaddition: A suspension of G-quadruplex nano-template G_4•Au@Fe_3O_4 (10 μL) in 20 μL Tris·KCl buffer (100 mM, pH 7.4) was taken in a 1.5 mL eppendorf tube. Alkynes **1a-c** (0.6 μM) were then added to the mixture, followed by the addition of 2.4 μM of each azide **2**-**12**. Then the mixture was stirred at rt for 6 days. The separation procedures were optimized to obtain the lead compounds.

Optimization of TGS using G-quadruplex nano-template: After 6 days of incubation, the reaction mixture was treated with 8M LiCl and heated to 65 °C to separate the products from the DNA nano-template. The nanoparticles were separated by using an external magnet and the resulting supernatant was analyzed by ESI-

MS and HPLC. The MS analysis showed that the supernatant contained a mixture of newly generated triazole products and unreacted azide and alkyne building blocks. The formation of triazole products indicated that the DNA-MNPs could promote the coupling of mutually compatible alkyne and azide fragments by bringing them in proximity. The HPLC chromatogam of the supernatant however showed an inseparable complex mixture of compounds with overlapping peaks (**Supplementary Figure 6a)**. Additionally, the Li ions destabilize the quadruplex confirmation and therefore the separated G_4●Au@Fe_3O_4 MNPs could not be reused for another round of azide-alkyne cycloaddition. Subsequently, the purification protocol was modified by heating the mixture at 65 °C without adding LiCl. However, the lead compounds could not be identified as overlapping peaks were obtained in HPLC analysis (**Supplementary Figure 6b)**.

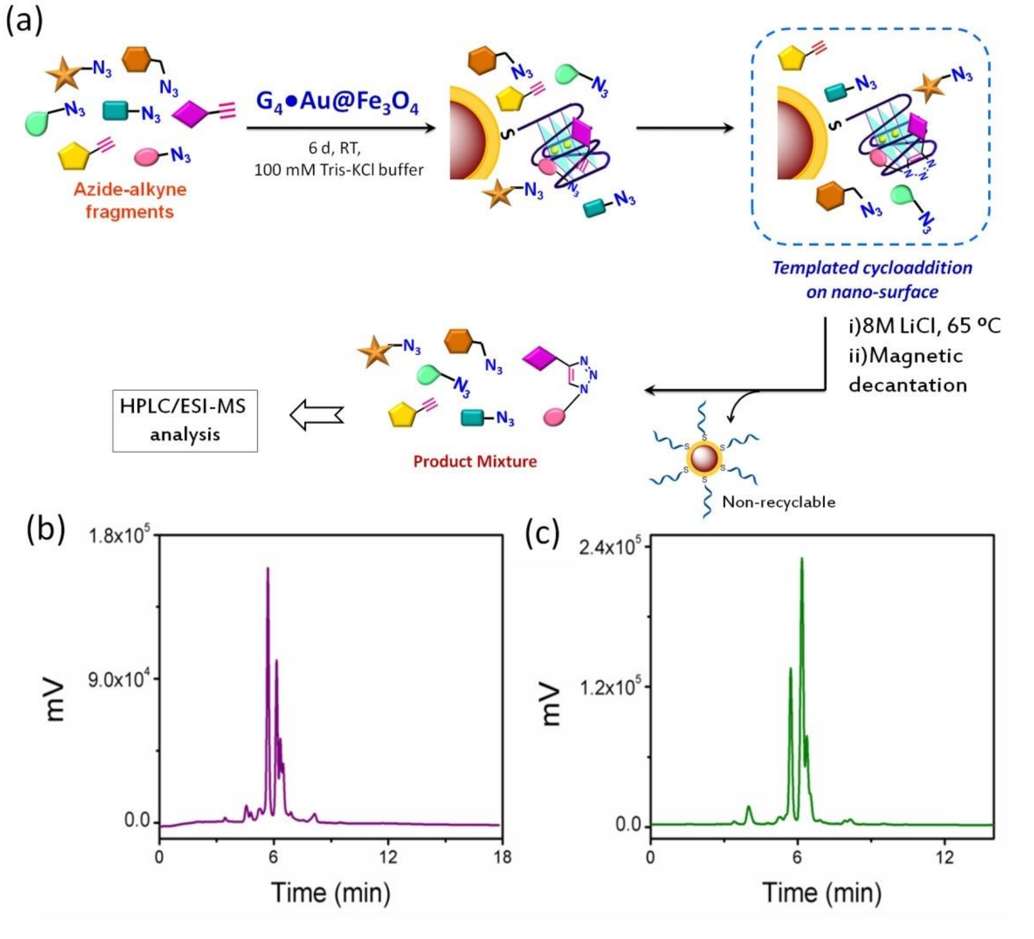

Supplementary Figure 6. Optimization of TGS using G_4●Au@Fe_3O_4 nano-template. (**a**) Schematic representation of the separation process to identify the triazole lead compounds. (**b**) HPLC chromatogram of the supernatant collected following the above mentioned separation protocol with **G_4●Au@Fe_3O_4**; giving mixtures of unreacted building blocks along with the newly generated products. (**c**) The supernatant collected by using the same protocol but without adding LiCl also gave overlapping peaks in HPLC chromatogram

Modified Separation protocol: In another set of experiment, after 6 days of reaction, G_4●Au@Fe_3O_4 nanoparticles were separated from the reaction mixture using a magnet and washed thrice with 100 mM Tris.KCl buffer, pH 7.4 (100 μL) to remove the unreacted starting materials. Afterwards, the nanoparticles were dispersed in 100 mM Tris.KCl buffer, pH 7.4 (50 μL) and the dispersion was then heated for 5 min at 65 °C and separated

instantly. The supernatant contained the triazole lead compounds, which were identified by HPLC and ESI-MS spectroscopy.

Time-dependent cycloaddition using DNA nano-template: A mixture of alkynes **1a-c** (0.6 μM) and azides **2**-**12** (2.4 μM of each) was stirred in the presence of G_4●Au@Fe_3O_4 for two and four days. The product distribution was analyzed by HPLC and ESI-MS spectroscopy (**Supplementary Figure 7**) and compared with the HPLC chromatogram of 6 d reaction.

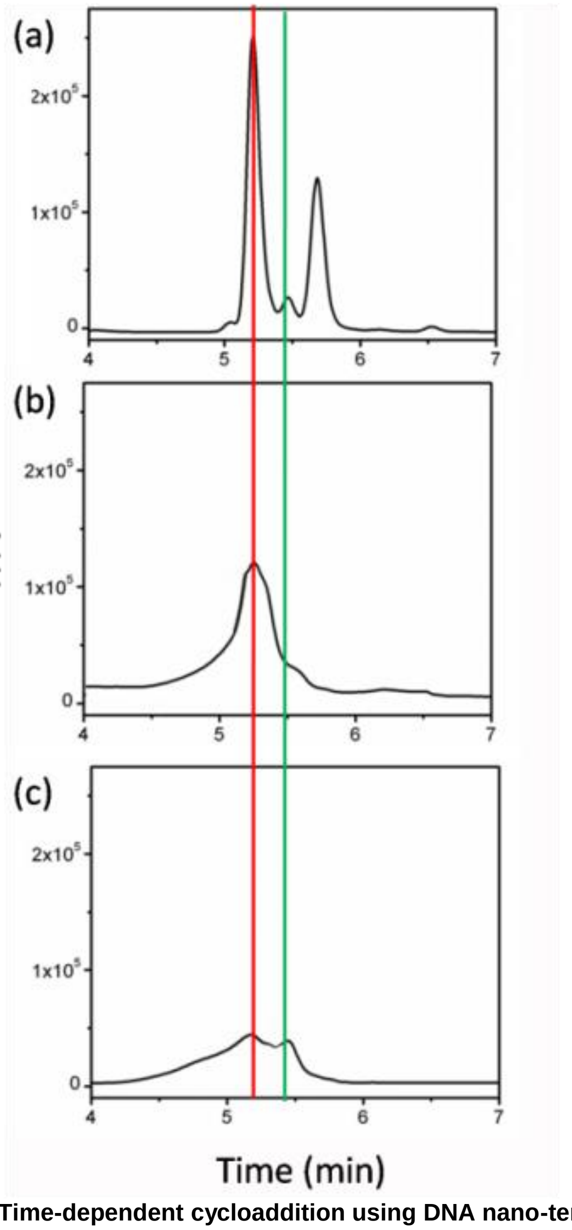

Supplementary Figure 7. Time-dependent cycloaddition using DNA nano-template. HPLC chromatogram of the supernatant collected from the azide-alkyne cycloaddition in the presence of **G_4●Au@Fe_3O_4** after (**a**) 6 days, (**b**) 4 days and (**c**) 2 days (as per the modified separation protocol).

Templated azide-alkyne cycloaddition using *ds*DNA●Au@Fe_3O_4: A suspension of duplex DNA nano-template *ds*DNA●Au@Fe_3O_4 (10 μL) in 20 μL Tris·KCl buffer (100 mM, pH 7.4) was taken in a 1.5 mL eppendorf tube. Then, alkynes **1a-c** (0.6 μM) were added to the mixture, followed by the addition of 2.4 μM of each azide **2**-**12**.

Then the mixture was stirred at rt for 6 days. The aforementioned modified Separation Protocol was used to separate the *ds*DNA nano-template and the generated triazole product (**Supplementary Figure 8**).

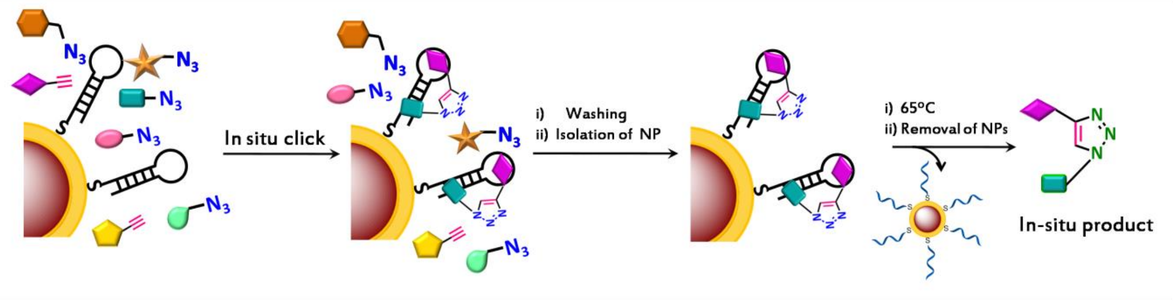

Supplementary Figure 8. Templated azide-alkyne cycloaddition using *ds*DNA●Au@Fe_3O_4. Schematic representation of *ds*DNA●Au@Fe_3O_4 catalysed azide-alkyne cycloaddition.

Determination of the regiochemistry of the triazole product Tz 1: The regiochemistry of the triazole compound **Tz 1**, generated by G_4●Au@Fe_3O_4 was determined by comparing the HPLC traces of the templated cycloaddition (*in situ* reaction) with the typical thermal and Cu-(I) catalyzed reactions between alkyne **1a** and azide **11** (**Supplementary Figure 9**).

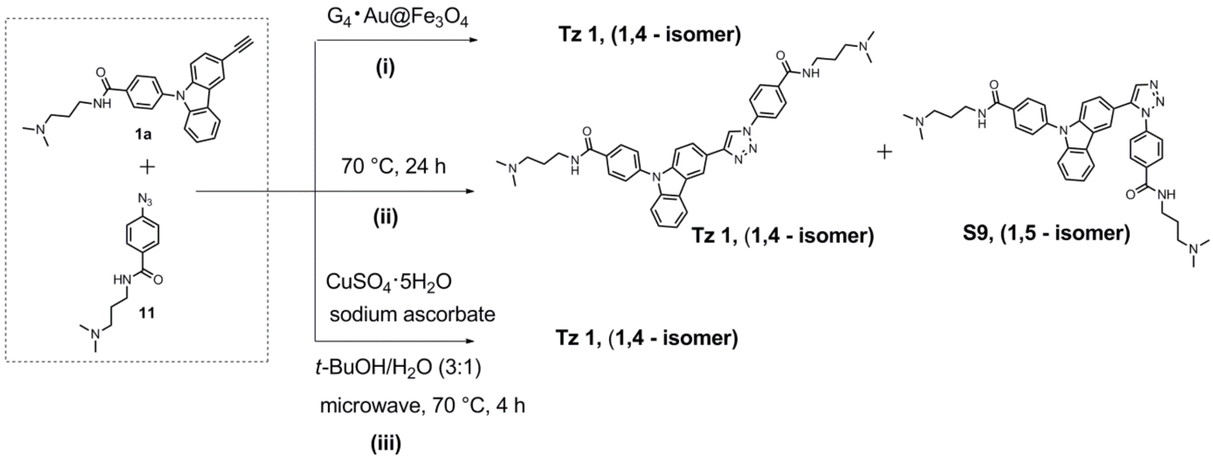

Supplementary Figure 9. Cycloaddition of alkyne **1a** and azide **11** using (**i**) G_4●Au@Fe_3O_4, (**ii**) thermal and (**iii**) Cu(I) catalysed conditions.

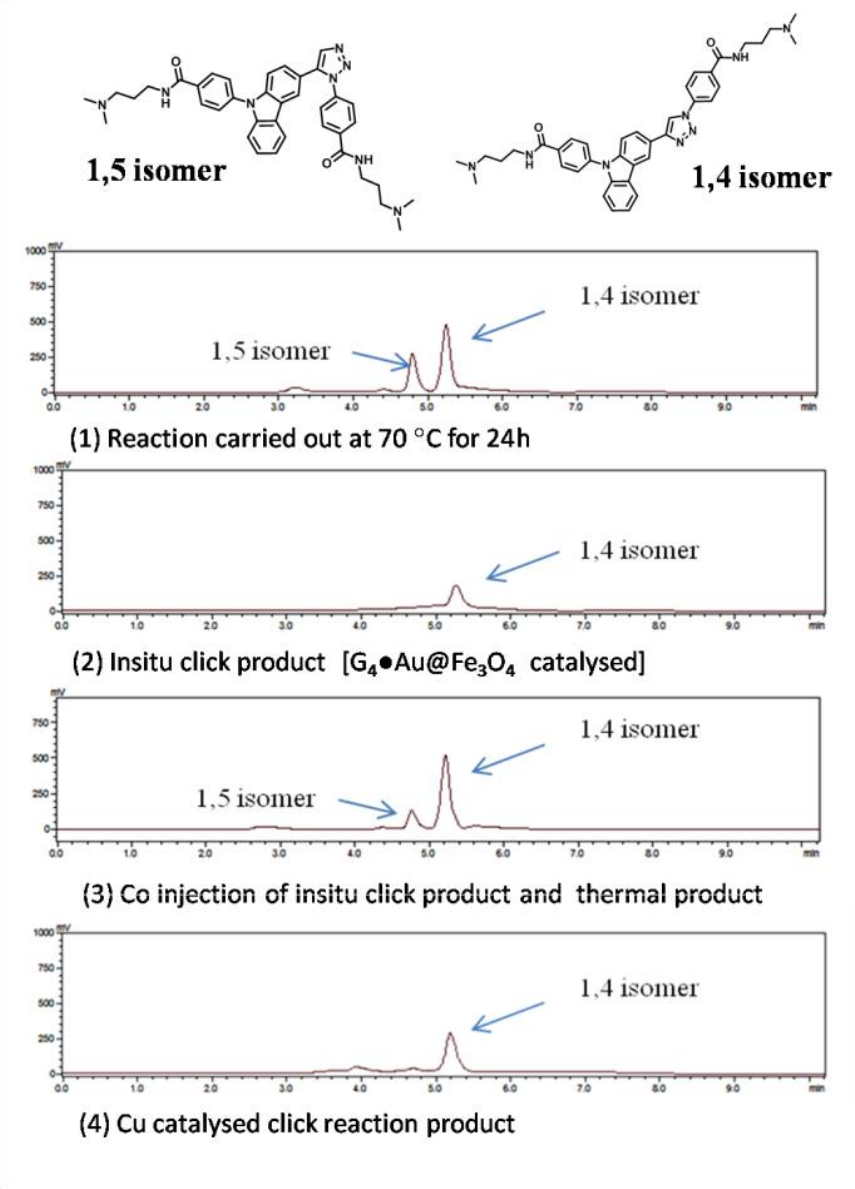

Supplementary Figure 10. Regiochemistry determination for compound Tz 1. Chromatographic traces for the 1,3-dipolar cycloaddition between alkyne **1a** and azide **11** obtained under different conditions.

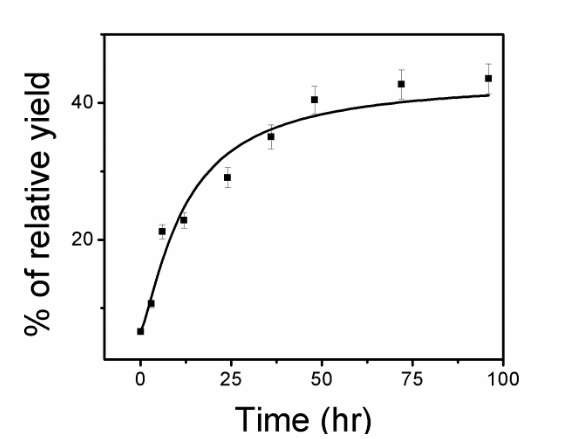

Supplementary Figure 11. Relative yield of Tz 1 from G_4●Au@Fe_3O_4 templated reaction. Yield as a function of the reaction time for TGS performed by G_4●Au@Fe_3O_4 nano-template with alkyne **1a** and azide **11.**

Recycling of *c-MYC* DNA nano-template: For each cycle, the recovered G_4•Au@Fe_3O_4 nano-template was used and TGS was performed with alkyne **1a-c** (0.6 μM each) and azide **2-12** (2.4 μM each). The reaction conditions were similar as mentioned in the modified separation protocol.

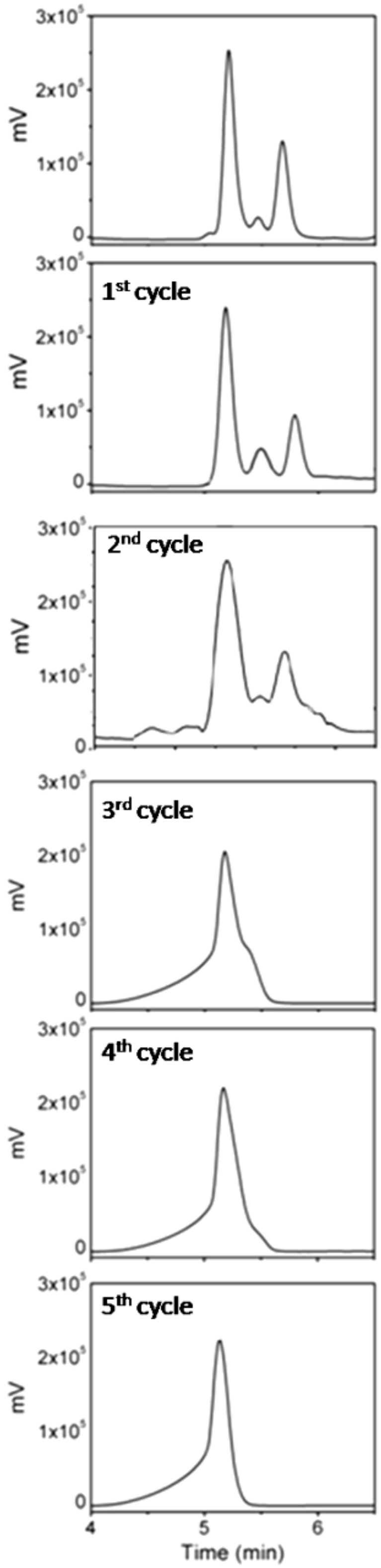

Supplementary Figure 12: Recycling of G_4•Au@Fe_3O_4 nano-template. HPLC chromatograms show the ability of recovered G_4•Au@Fe_3O_4 nano-template in promoting cycloaddition for five reaction cycles.

General procedure for the synthesis of triazole products (Tz 1-3) by Cu(I) catalyzed Huisgen cycloaddition (GP-1): Carbazole alkyne **1a** (65.7 mg, 0.166 mmol) was dissolved in a 2:1 mixture of *t*-BuOH/H_2O (4 mL). Copper (II) sulphate pentahydrate (4.1 mg, 0.0166 mmol) and sodium ascorbate (3.2 mg, 0.0166 mmol) were added and the solution was stirred for 10 min. The desired azide (**3, 7** & **11**) (1.2 × 0.166 mmol equiv.) was added separately and the mixture was heated for 4 h at 70 °C under microwave irradiation (**Supplementary Figure 13**). After cooling to the room temperature, the reaction mixture was concentrated. The crude product was purified by flash column chromatography (using CH_2Cl_2 (100%)-CH_2Cl_2/MeOH (10:1) - CH_2Cl_2/MeOH/NH_4OH (10:1:0.5) to provide the corresponding triazole products **Tz 1-3**.

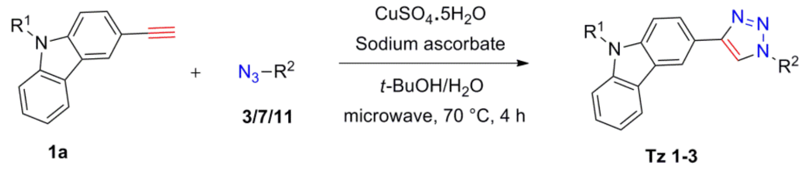

Supplementary Figure 13. General method for the synthesis of 1,4-substituted triazole products *via* Cu(I) catalysed azide-alkyne cycloaddition.

Analytical data of compounds:

Triazole derivative Tz 1: Following the **GP-1**, the reaction of the alkyne **1a** with azide **11** (49 mg) afforded **Tz 1** (47 mg, 64%) as a yellow solid. R_f = 0.15 (CH_2Cl_2/MeOH/NH_4OH (10:1:0.5); ^{1}H NMR (500 MHz, DMSO-d^6): δ 9.43 (s, 1H), 8.85 (s, 1H), 8.72-8.69 (m, 2H), 8.33 (d, *J* = 8.4, 1H), 8.16 (d, *J* = 8.4, 2H), 8.11 (s, 4H), 8.06 (d, *J* = 8.4, 1H), 7.79 (d, *J* = 8.4, 2H), 7.57 (d, *J* = 8.4, 1H), 7.51-7.47 (m, 2H), 7.36 (t, *J* = 8.4, 1H), 3.33 (4H, merged with water peak), 2.36-2.33 (m, 4H), 2.21-2.19 (12H, two single peak merged), 1.74-1.69 (m, 4H); ^{13}C NMR (100 MHz, DMSO-d^6): δ 165.3, 165.0, 148.2, 140.3, 139.0, 138.3, 134.3, 135.6, 129.1, 128.9, 126.8, 126.2, 124.1, 123.4, 122.9, 122.7, 120.7, 119.3, 118.8, 117.4, 110.3, 109.9, 56.8, 45.0, 39.5 (merged with DMSO peak) 37.7, 26.9; HRMS (ESI) Calcd for $C_{38}H_{43}N_8O_2$ $[M+H]^+$ 643.3509, Found 643.3502.

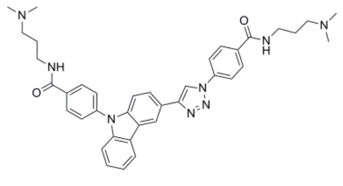

Tz 1

Triazole derivative Tz 2: Following the **GP-1**, the reaction of the alkyne **1a** with azide **3** (26 mg) afforded **Tz 2** (60 mg, 69%) as a yellow viscous liquid. R_f = 0.35 (CH_2Cl_2/MeOH/NH_4OH (10:1:0.5); ^{1}H NMR (500 MHz, DMSO-d^6): δ 8.72 (s, 2H), 8.60 (s, 1H), 8.30 (d, *J* = 8.4, 1H), 8.13 (d, *J* = 8.4, 2H), 7.95 (d, *J* = 8.4, 1H), 7.76 (d, *J* = 8.4, 2H), 7.52-7.47 (m, 3H), 7.34 (t, *J* = 7.5, 1H), 4.44 (t, *J* = 6.7, 2H), 3.35 (2H, merged with water peak), 2.37 (s, 2H), 2.23-2.17 (m, 12H), 2.04 (t, *J* = 6.7, 2H), 1.79-1.71 (m, 4H); ^{13}C NMR (125 MHz, DMSO-d^6): δ 165.6, 147.1 140.3, 139.5, 139.2, 133.5, 129.2, 126.8, 126.3, 124.1, 123.6, 123.5, 123.0, 120.8, 117.2, 110.2, 110, 47.8, 45.1, 44.9, 40.5(merged with DMSO peak), 37.6, 27.7, 26.9; HRMS (ESI) Calcd for $C_{31}H_{38}N_7O$ $[M+H]^+$ 524.3138, Found 524.3133.

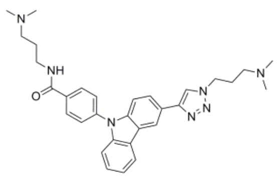

Tz 2

Triazole derivative Tz 3: Following the **GP-1**, the reaction of the alkyne **1a** with azide **7** (27 mg) afforded **Tz 3** (67 mg, 59%) as a brown solid. R_f = 0.42 (CH_2Cl_2/MeOH/NH_4OH (10:1:0.5); ^{1}H NMR (500 MHz, DMSO-d^6): δ

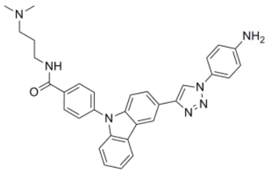

Tz 3

9.06 (s, 1H), 8.81 (s, 1H), 8.78 (s, 1H), 8.32 (d, *J* = 8.6, 1H), 8.17 (d, *J* = 8.6, 2H), 8.04 (d, *J* = 8.6, 1H), 7.58-7.48 (m, 5H), 7.37 (t, *J* = 8.6, 1H), 6.74 (d, *J* = 8.6, 2H), 5.52 (s, 2H), 3.33 (2H, merged with water peak), 2.71 (s, 2H), 2.47 (s, 6H), 1.83 (s, 2H); ^{13}C NMR (100 MHz, DMSO-d^6): δ 165.6, 149.4, 147.5, 140.3, 139.5, 139.2, 133.3, 129.2, 126.7, 126.2, 126.1, 124.1, 123.4, 122.9, 121.4, 120.7, 118.5, 117.3, 113.9, 110.2, 109.9, 55.7, 43.5, 36.7, 28.6; HRMS (ESI) Calcd for $C_{32}H_{32}N_7O$ $[M+H]^+$ 530.2628, Found 530.2624.

HPLC method: HPLC analyses were performed using SHIMADZU, SPD-20A system equipped with a Waters Spherisorb® 5.0 *µm* ODS2 column 4.6 x 250 mm using 254 nm detection wavelength and 2 µL injection volume. Flow rate was 0.5 *mL*/min CH_3CN/H_2O (90:10) in 0.1% TFA over 20 minutes.

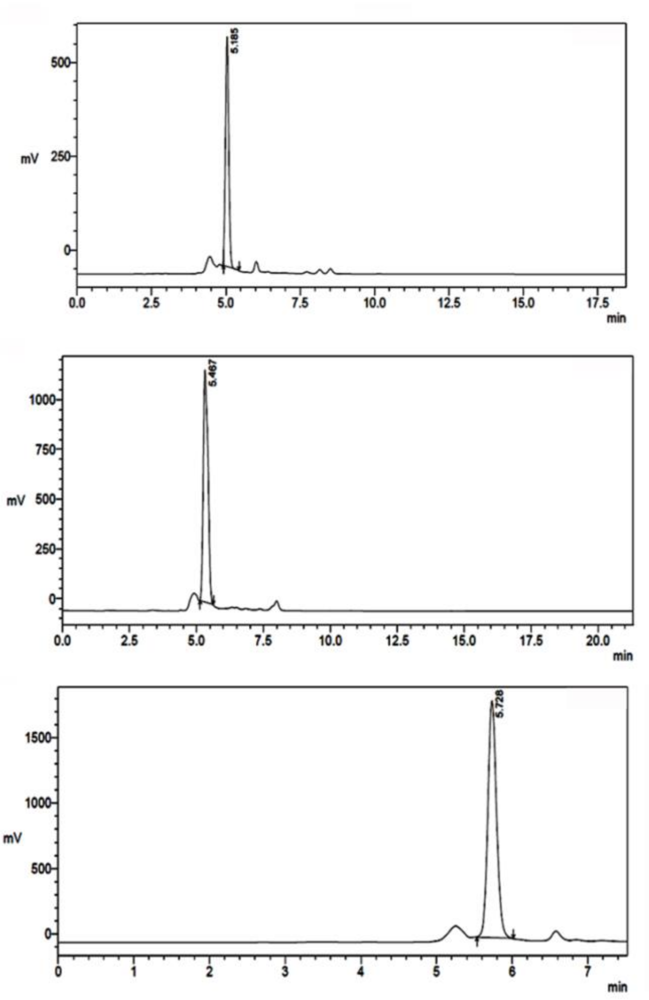

Supplementary Figure 14. HPLC chromatograms of triazole products **Tz 1-3**.

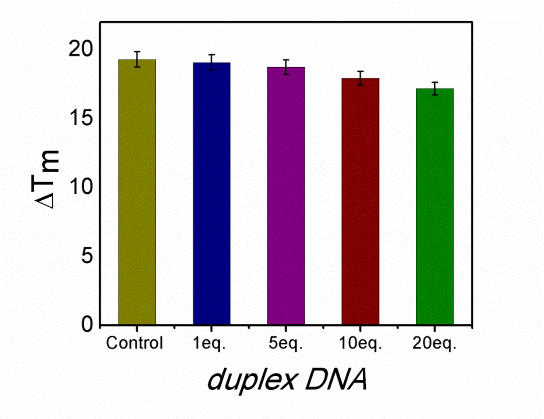

Supplementary Figure 15. Competitive FRET-melting experiment. ΔT_m values of *c-MYC* G-quadruplex DNA (100 nM) in the presence of 1 μM **Tz 1** and increasing amount of unlabeled duplex DNA competitor (100 nM, 500 nM, 1.0 μM and 2.0 μM). (n = 3, ±s.e.m.)

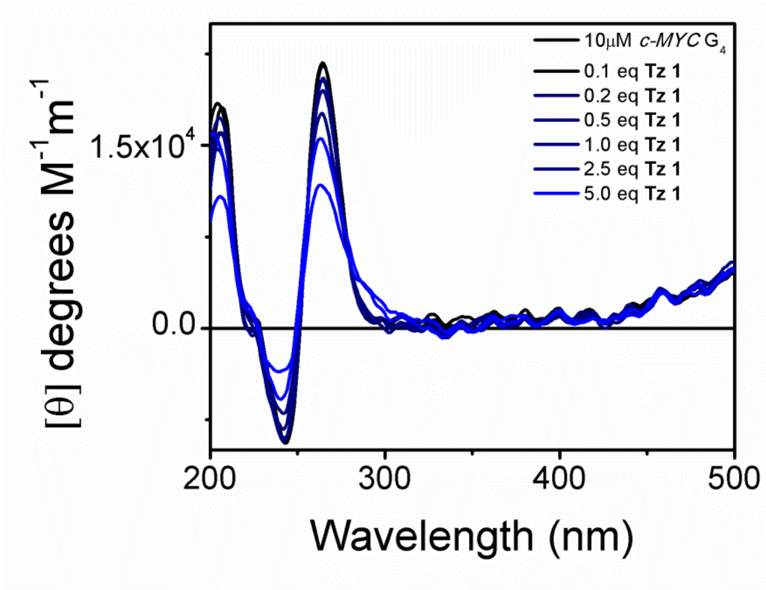

Supplementary Figure 16. CD spectra of *c-MYC*-G4:Tz 1 complex. CD spectra of *c-MYC* DNA (10 μM) in 100 mM Tris.KCl buffer (pH 7.4) titrated with 0-5 eq of **Tz 1**.

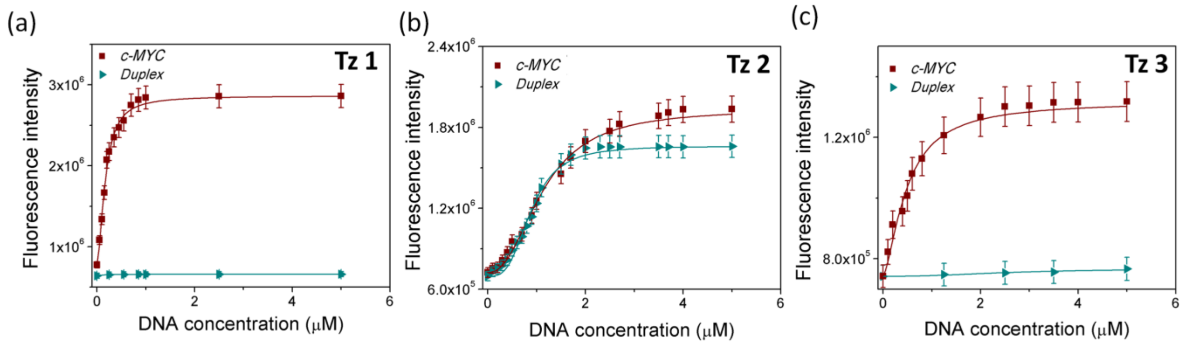

Supplementary Figure 17. Fluorescence response curves of Tz 1-3. Fluorescence responses of **Tz 1**, **Tz 2** and **Tz 3** (1 μM) with the stepwise addition of *c-MYC* G-quadruplex and duplex DNA in 100 mM Tris-KCl buffer, pH 7.4. (Compound **Tz 1**: λ_{ex} = 280 nm, λ_{em} = 493 nm; Compound **Tz 2**: λ_{ex} =285 nm, λ_{em} = 440 nm and Compound **Tz 3**: λ_{ex} = 278 nm, λ_{em} = 500 nm). (n = 3, ± s.e.m.)

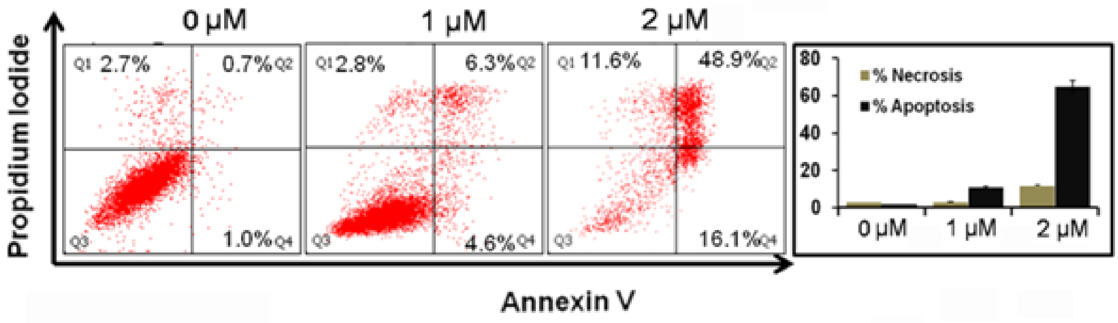

Supplementary Figure 18. Apoptotic cell death induced by Tz 1. Compound **Tz 1** induces significant apoptosis after 24 h treatment, as seen by the flow cytometric analysis of FITC-Annexin V/PI stained control and treated cells. Bar diagram shows percentage of apoptotic and necrotic HCT116 cells upon treatment with **Tz 1**. (n = 3, ± s.e.m.)

All the experiments were performed in triplicates and the best results were represented.

NMR spectra of compounds.

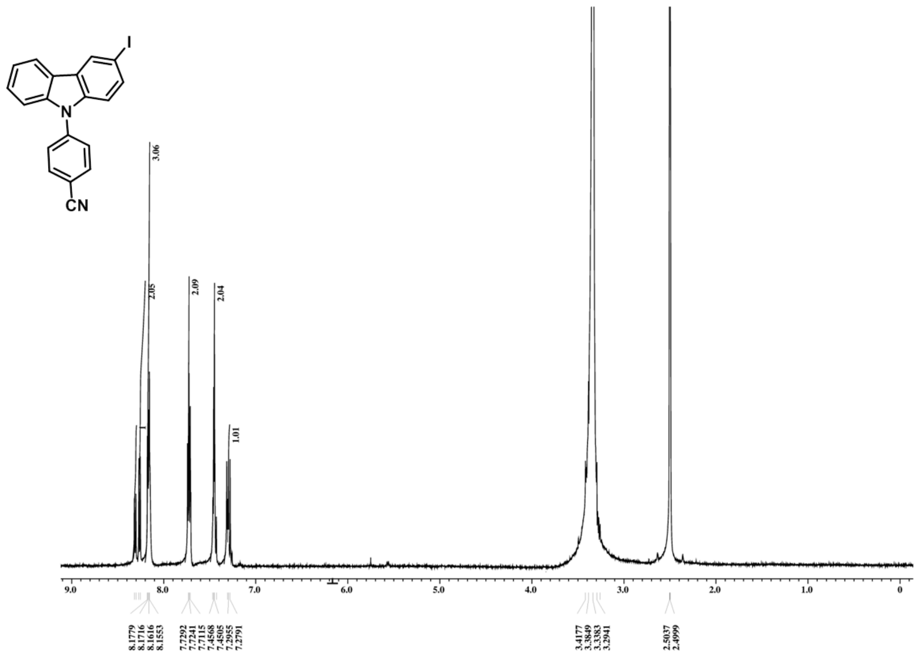

Supplementary Figure 20. ^{1}H NMR spectrum of 4.

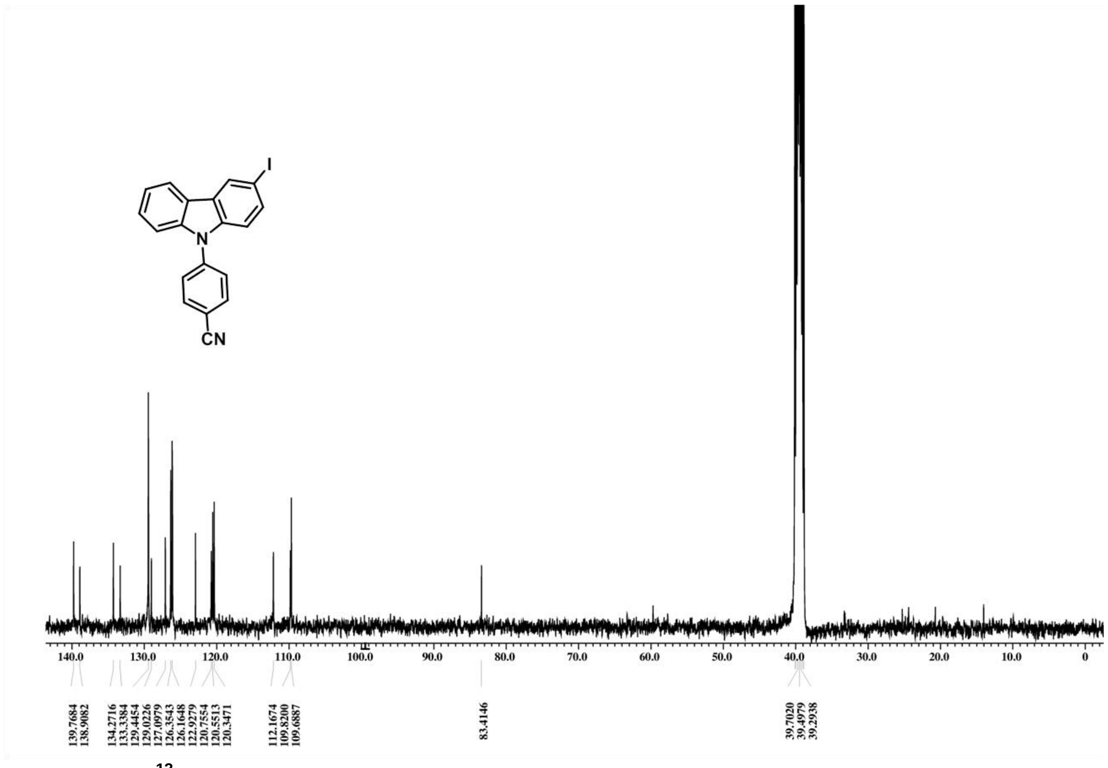

Supplementary Figure 21. ^{13}C NMR spectrum of 4.

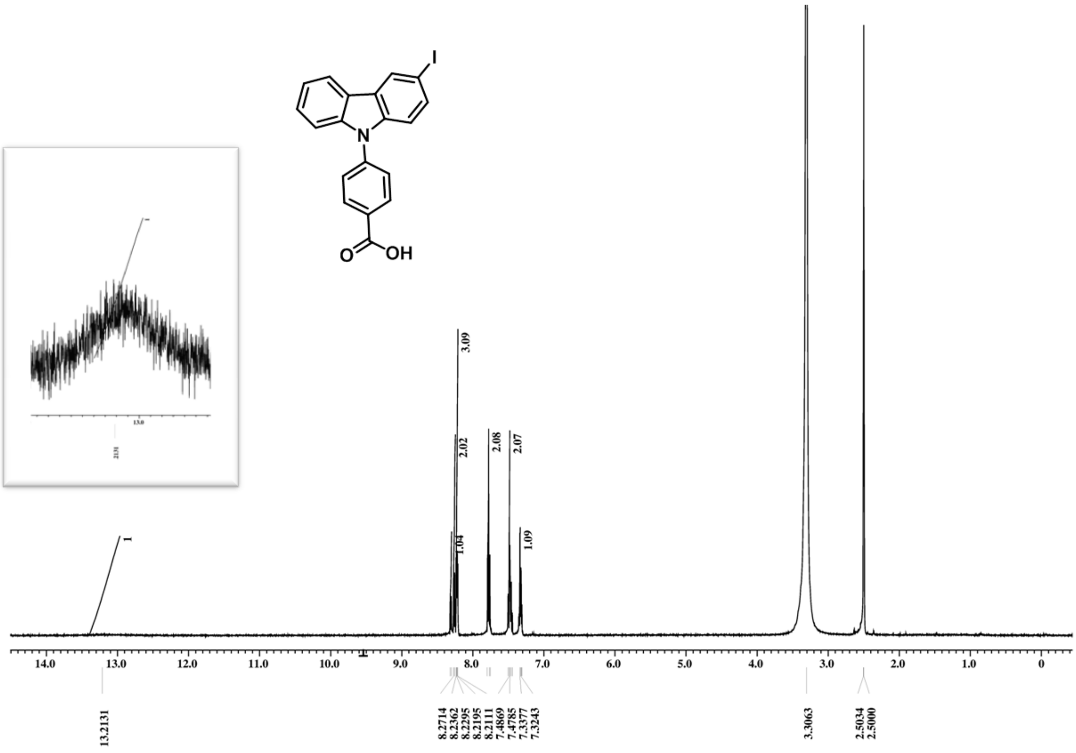

Supplementary Figure 22. ^{1}H NMR spectrum of 5.

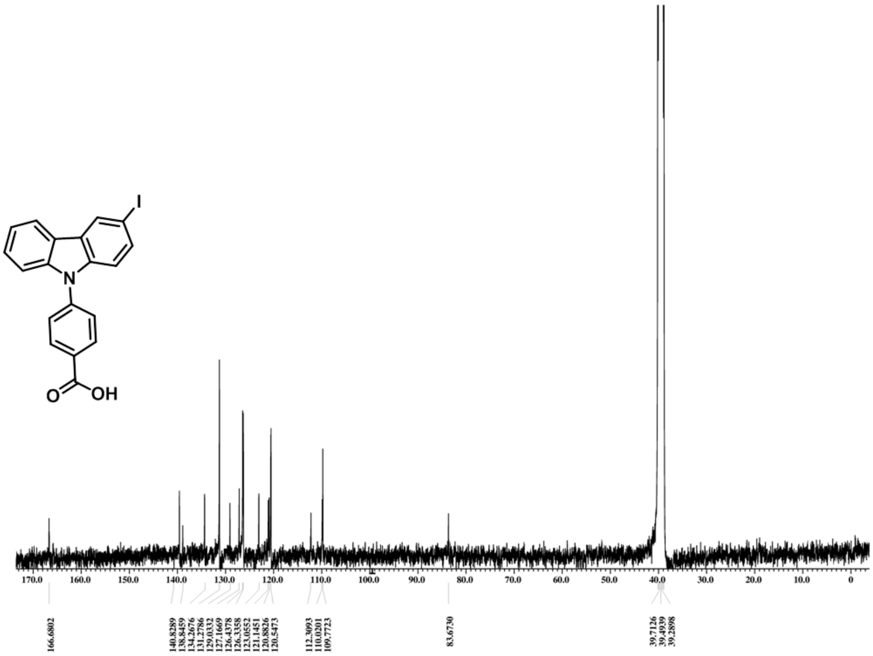

Supplementary Figure 23. ^{13}C NMR spectrum of 5.

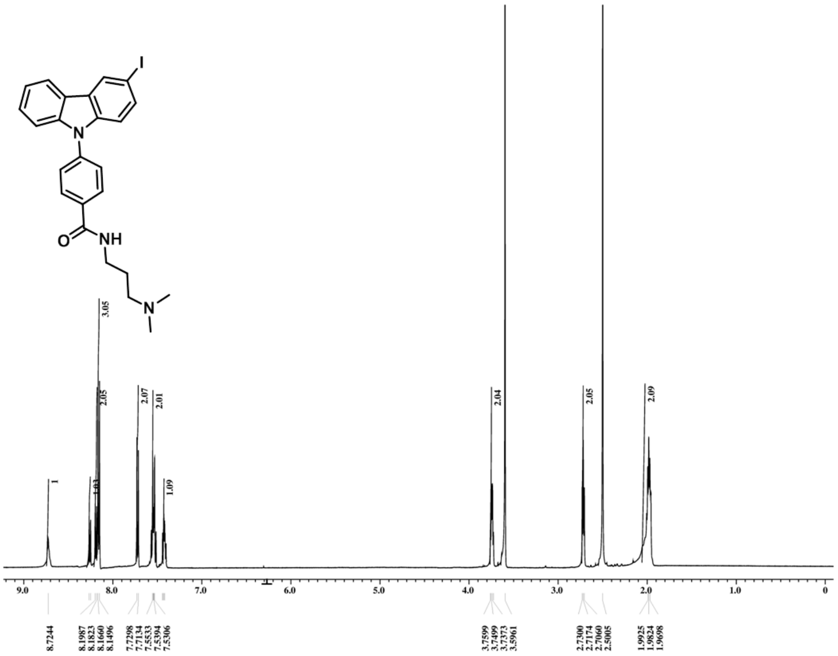

Supplementary Figure 24. ^{1}H NMR spectrum of 7.

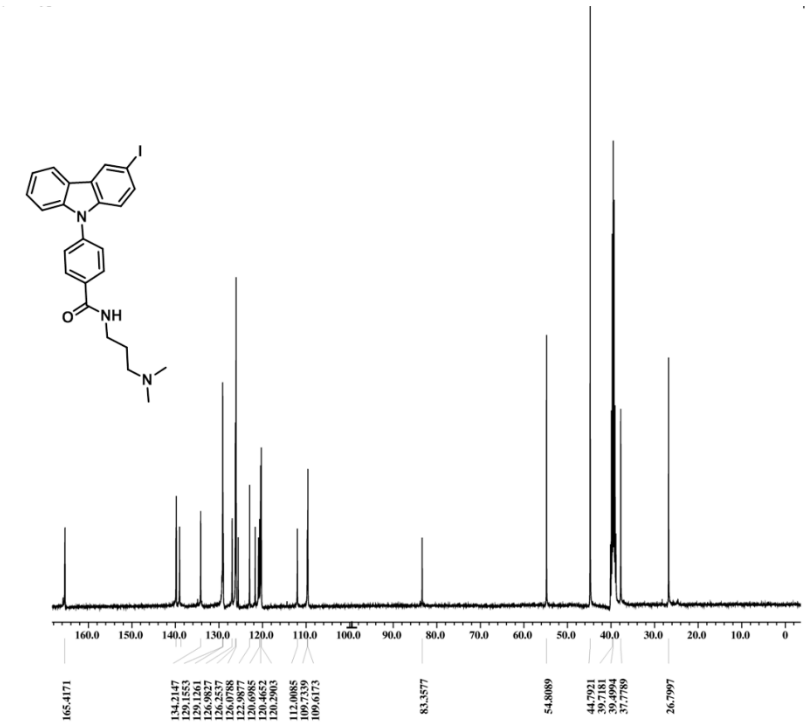

Supplementary Figure 25. ^{13}C NMR spectrum of 7.

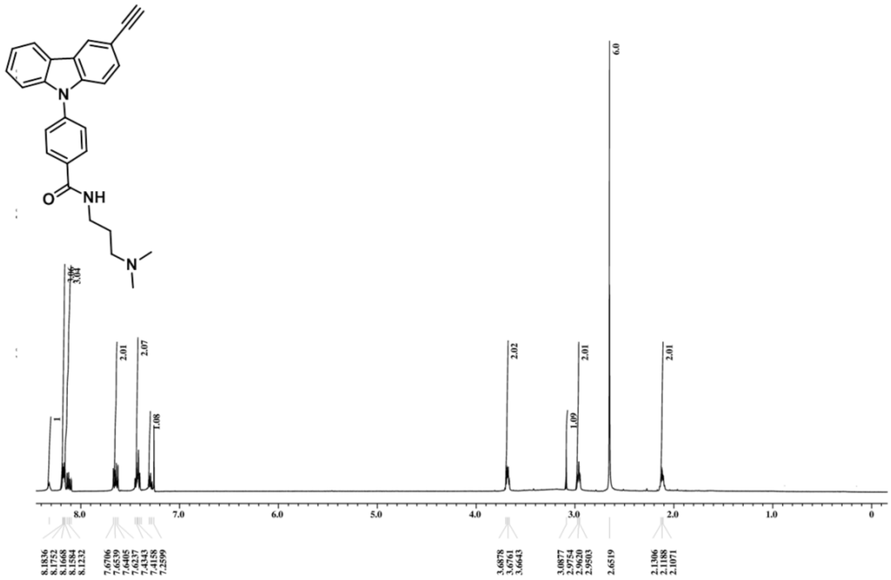

Supplementary Figure 26. ^{13}**H NMR spectrum of 1a.**

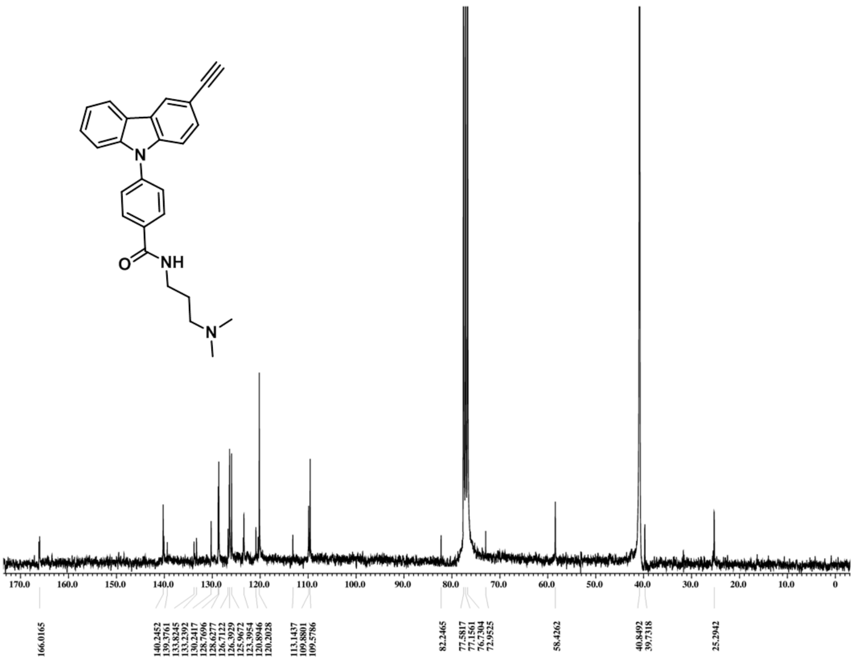

Supplementary Figure 27. ^{13}**C NMR spectrum of 1a.**

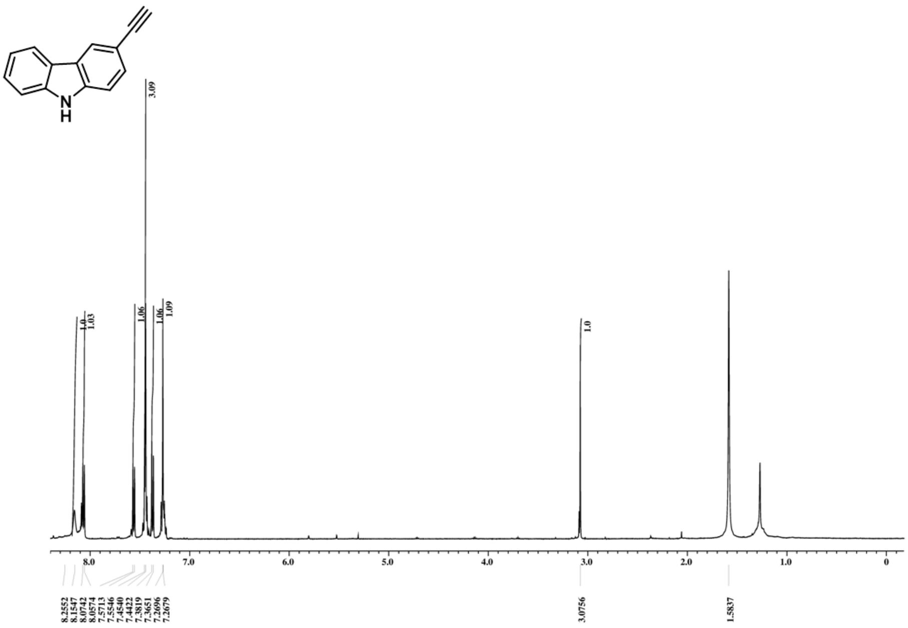

Supplementary Figure 28. ^{1}**H NMR spectrum of 1b.**

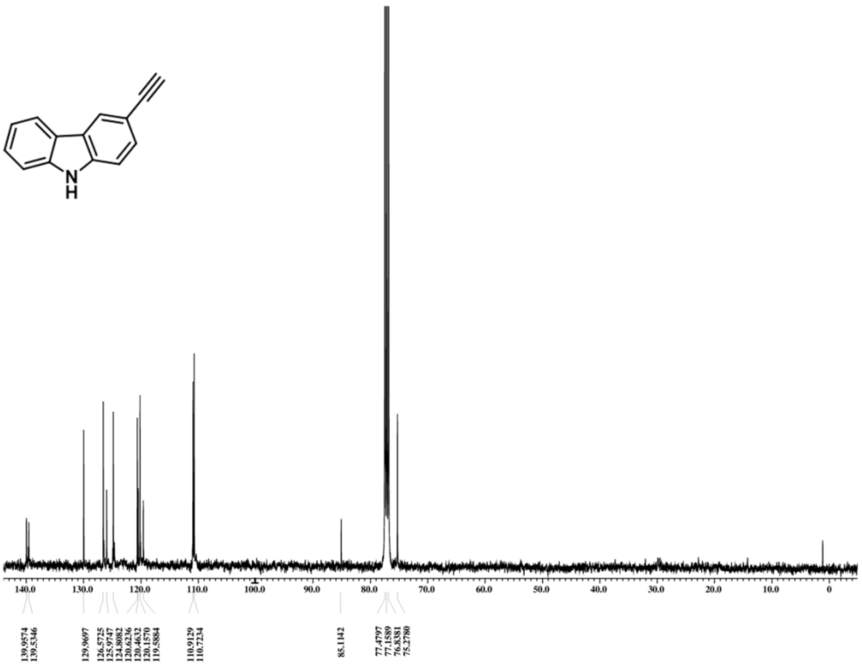

Supplementary Figure 29. ^{13}**C NMR spectrum of 1b.**

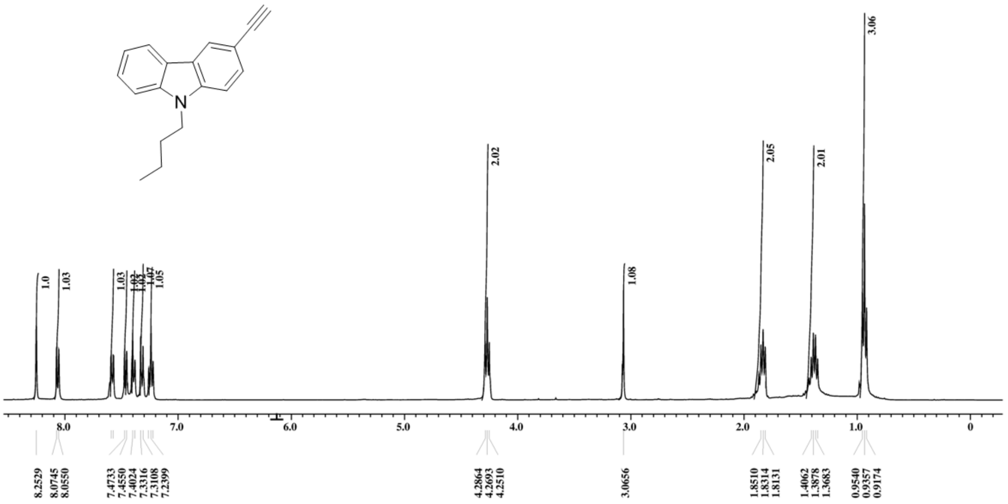

Supplementary Figure 30. ^{1}H NMR spectrum of 1c.

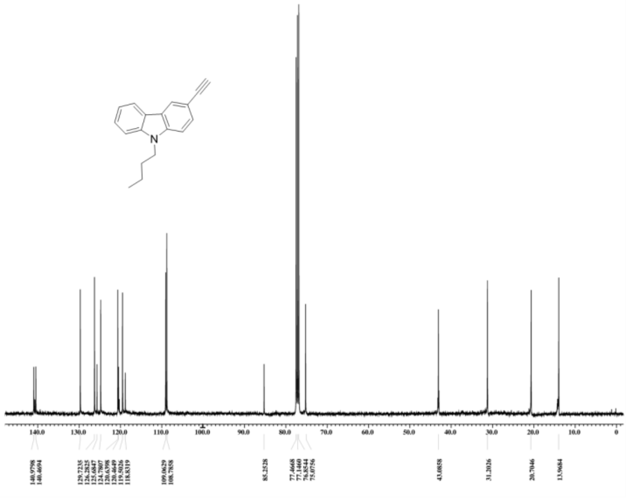

Supplementary Figure 31. ^{13}C NMR spectrum of 1c.

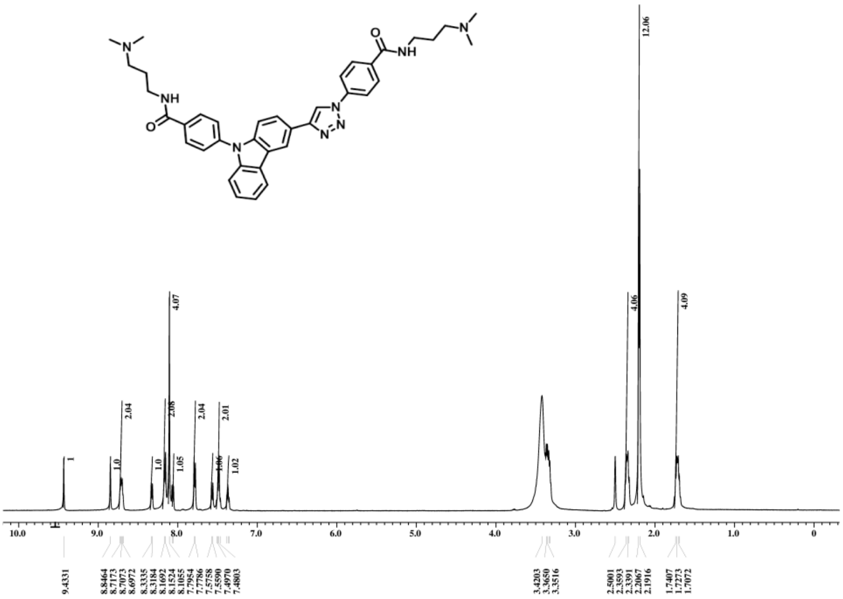

Supplementary Figure 32. ^{1}H NMR spectrum of Tz 1.

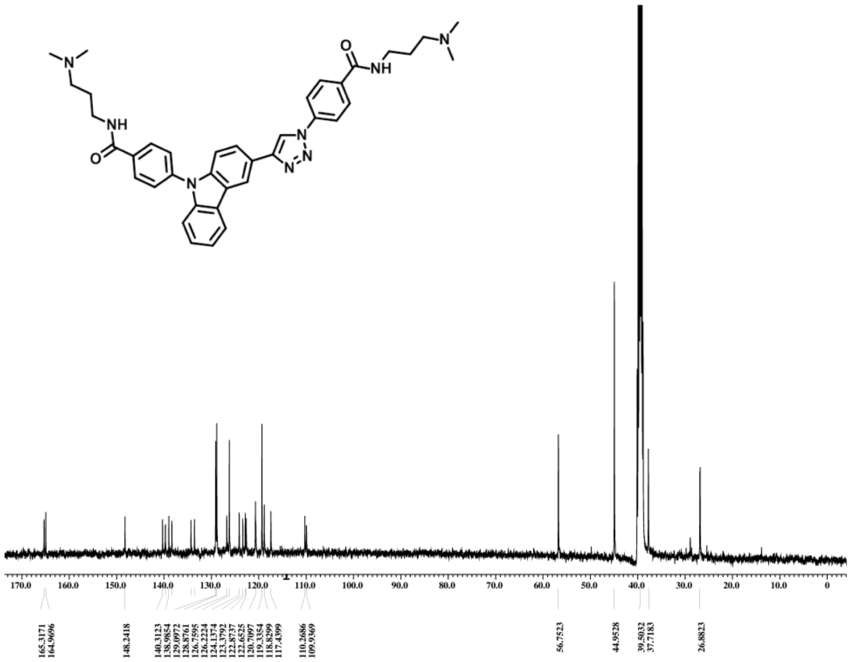

Supplementary Figure 33. ^{13}C NMR spectrum of Tz 1.

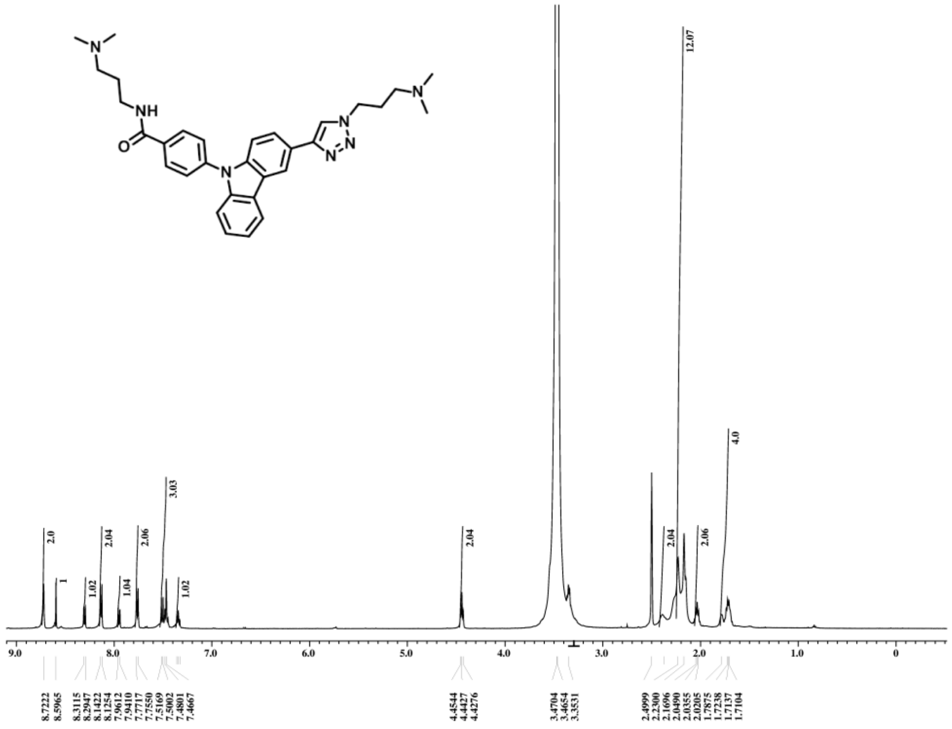

Supplementary Figure 34. ^{1}H NMR spectrum of Tz 2.

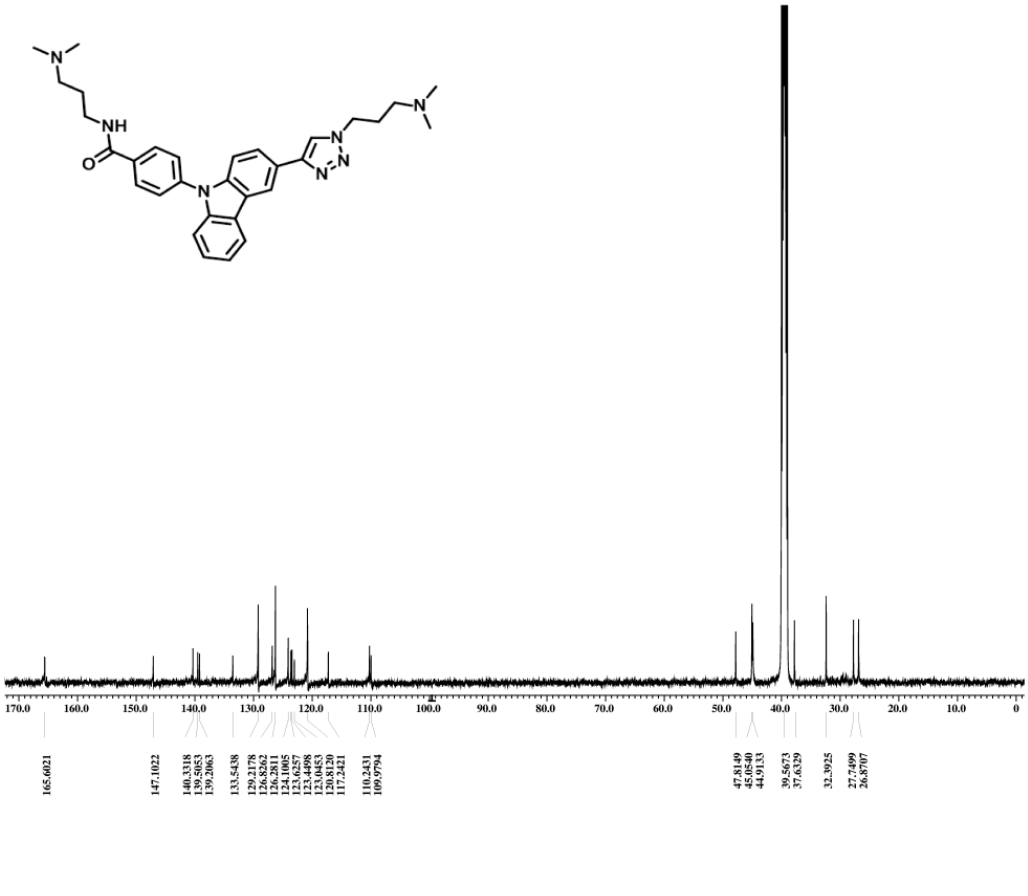

Supplementary Figure 35. ^{13}C NMR spectrum of Tz 2.

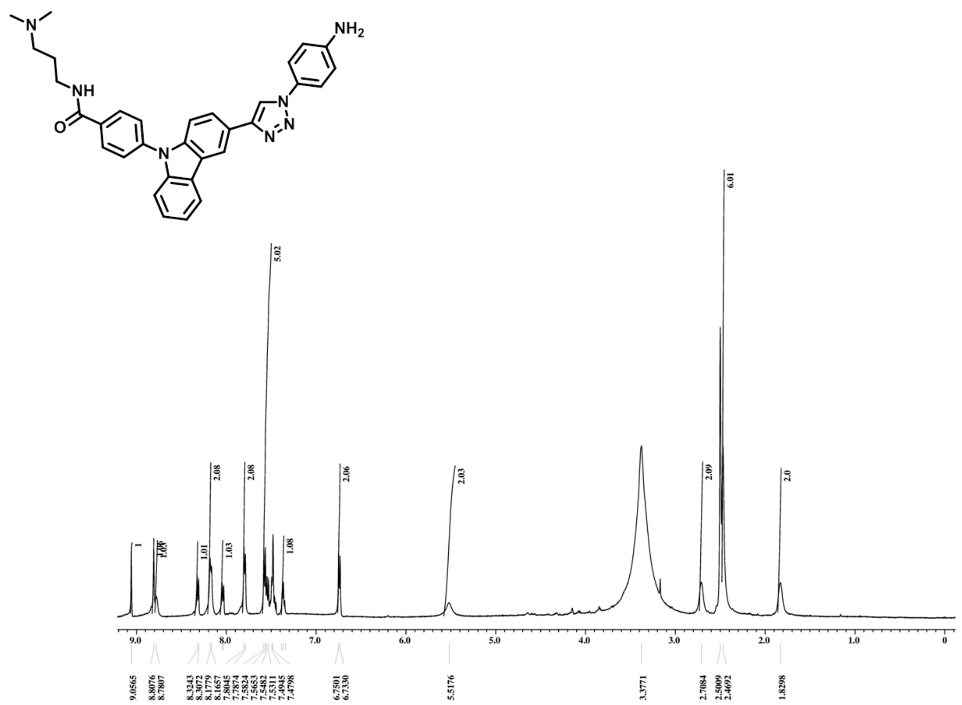

Supplementary Figure 36. ^{1}H NMR spectrum of Tz 3.

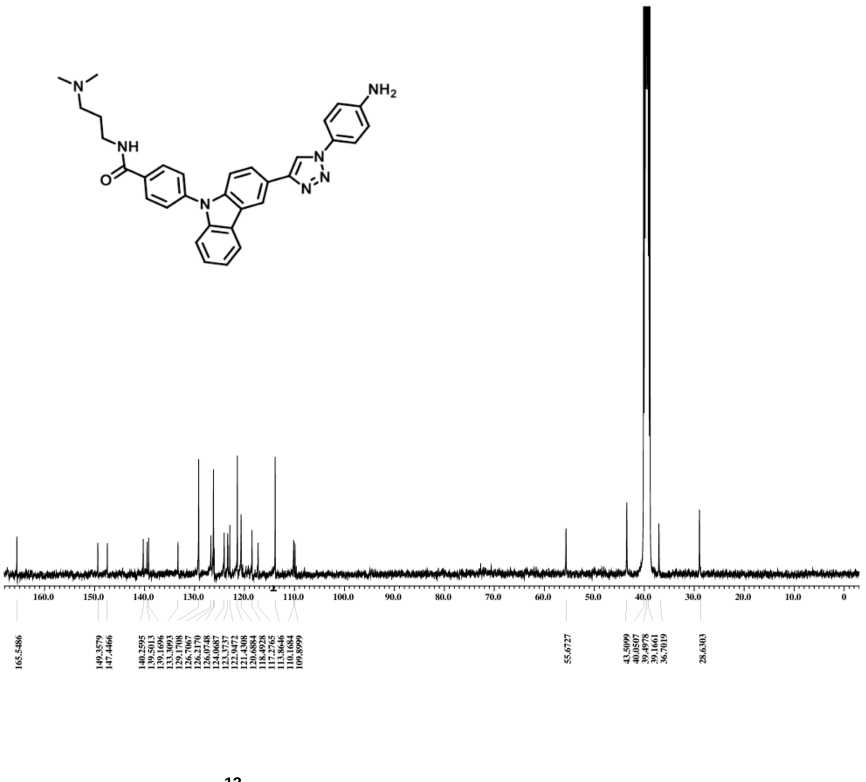

Supplementary Figure 37. ^{13}C NMR spectrum of Tz 3.

Supplementary References.

1. Song, B. J. *et al.* A Desirable Hole-Conducting Coadsorbent for Highly Efficient Dye-Sensitized Solar Cells through an Organic Redox Cascade Strategy. *Chem-Eur J* **17**, 11115-11121 (2011).

2. Wu, Y. B., Guo, H. M., James, T. D. & Zhao, J. Z. Enantioselective Recognition of Mandelic Acid by a 3,6-Dithiophen-2-yl-9H-carbazole-Based Chiral Fluorescent Bisboronic Acid Sensor. *J Org Chem* **76**, 5685-5695 (2011).
